# Plasma metabolomic profiles and immune responses of piglets after weaning and challenge with *E. coli*

**DOI:** 10.1186/2049-1891-5-17

**Published:** 2014-03-14

**Authors:** Sugiharto Sugiharto, Mette S Hedemann, Charlotte Lauridsen

**Affiliations:** 1 Department of Animal Science, Faculty of Science and Technology, Aarhus University, AU-Foulum, Tjele 8830, Denmark; 2 Faculty of Animal Science and Agriculture, Diponegoro University, Semarang, Central Java 50275, Indonesia

**Keywords:** *E. coli* F18, Immunity, Metabolite, Piglet, Weaning

## Abstract

**Background:**

The processes of weaning and exposure to pathogenic bacteria induce stress responses, which may alter the metabolism. In this study, we investigated the changes in plasma metabolites and immune responses in piglets in response to the stress induced by weaning and *Escherichia coli* challenge.

**Results:**

Fecal dry matter decreased (*P* = 0.003) and nearly half (44.4%) the piglets developed diarrhea on day 2 and 3 postweaning. The concentration of plasma immunoglobulin A was higher (*P* < 0.001) on day 11 postweaning than on day 0 or 4 postweaning. The levels of white blood cells increased continuously (*P* < 0.001) from day 0 to day 11 postweaning. Differences in the percentages of neutrophils (*P* = 0.029) and lymphocytes (*P* = 0.022) were seen, but the neutrophil/lymphocyte ratio did not differ in the period after weaning. A clear separation of the metabolomic profile data for day 0 and day 4 postweaning was observed with a principal components analysis (PCA) scores plot, and the data for day 11 were located between those for day 0 and day 4 postweaning. The plasma levels of proline, taurine, and carnitine were higher, whereas those of betaine, creatine, L-arginine and acetylcarnitine were lower on day 4 postweaning than on day 0. Levels of lysophosphatidylcholine and phosphatidylcholine were either higher or lower after weaning, depending on the chain lengths or characters of these metabolites.

**Conclusions:**

Our results show a clear separation in the plasma metabolomic profiles of piglets that corresponded to the fecal responses to stress on the piglets induced by weaning or exposure to a pathogen (*E. coli*). These plasma metabolite profiles suggest that the challenges induced proinflammatory responses in the piglets, resulting in postweaning diarrhea, which was associated with higher concentrations of IgA in the plasma.

## Introduction

In modern pig production, weaning is one of the most critical periods during the life of piglets [1] because they are exposed to several stressors. Together with a number of nutritional changes (from sow’s milk to solid feed), social changes (mixing with different litters and loss of the sow), and environmental changes (moving from the farrowing unit to the nursery pen), the piglets are exposed to various pathogens in their feed and the environment just as the passive immunity conferred by the antibodies in the sow’s milk have diminished [2,3]. For these reasons, weaning has been implicated in the induction of stress responses and an increased susceptibility to infection [3,4].

Changes in the microbial community in the gut may translate to an instability (imbalance) in the gut microbial ecosystem, leading to intestinal disorders [5,6]. Beside weaning-related stress, another factor responsible for this unbalanced gut microbiota is the presence of enterotoxigenic *Escherichia coli* (ETEC) in the guts of piglets [7]. Together with the weaning stress and the change from sow’s milk to a cereal-based diet, changes in the microbial composition of the gut during weaning have been suggested to affect the metabolism of piglets [8-10]. Similarly, the regulation of their metabolism may be altered when weaned piglets experience intestinal distress [3]. Recently, metabolomic analysis has been introduced as a method to study the metabolism of weaned piglets [9]. With this approach, we studied the metabolomic profiles of postweaning piglet plasma based on liquid chromatography–mass spectrometry (LC–MS) metabolomic data.

The first week after weaning is usually considered the most stressful period for piglets and is associated with intestinal disorders and the development of postweaning diarrhea (PWD) [3,11]. To establish a pig model of weaning-stress-induced intestinal disorder, the piglets in the present study were challenged with *E. coli* F18, a common porcine enteric pathogen, during the first 3 d after weaning. The purpose of the study was to obtain information on the changes in the plasma metabolites and immune responses of piglets in response to the stress induced by weaning and *E. coli* challenge.

## Materials and methods

### Experimental design

The animal experiment was conducted according to the license obtained from the Danish Animal Experiments Inspectorate, Ministry of Food, Agriculture and Fisheries, Danish Veterinary and Food Administration. The experiment was conducted over an 11-day period after weaning with 18 specific-pathogen-free piglets from three litters. The pigs were weaned at 28–34 d of age (body weight = 9.18 ± 1.35 kg; mean ± SD) from sows that tested positive for *E. coli* F18, and were transported from the farrowing unit to the nursery pen. Three pigs of similar weight were housed together in 1.45 m × 1.70 m pens with concrete flooring and sawdust bedding. The weaned piglets had free access to creep feed (without zinc oxide) and fresh water from the time of weaning onward. They were also provided with a milk replacer during the first 4 d after weaning. The composition and chemical analysis of the creep feed and milk replacer are shown in Table 1. The milk replacer was prepared with an automated wet feeder (Mambo Automix 25, Wit-Mambo Inc., Wommels, Netherlands), in which milk powder was automatically mixed with warm water (45°C) (approx. 150 g of milk powder in 1 L of water), and was fed to the piglets on a regular basis.

**Table 1:** Creep feed and milk replacer compositions

**Creep feed**^**a**^				**Milk replacer**^**b**^				
Ingredients^c^,%				Ingredients^c^,%	
Wheat		46.80		Skim milk powder	50.00
Barley		25.00		Whey protein powder	24.00
Soybean meal		19.00		Vegetable oil and fat	13.00
Protein concentrate		2.00		Sugar	8.00
Palm fat		1.40		Wheat flour	4.00
Other^d^		5.80			
Chemical analysis^c^,%				Chemical analysis^c^,%	
Crude fat		3.50		Crude fat	14.00
Crude protein		18.30		Crude protein	23.00
Crude ash		5.30		Crude ash	7.00
Sodium		0.20		Sodium	0.47
Calcium		0.88		Calcium	0.75
Phosphorus		0.54		Phosphorus	0.68
Lysine		1.19		Lysine	2.17
Methionine		0.36		Methionine and cysteine	0.92

^a^Creep feed was produced by Dansk Landbrugs Grovvareselskab Amba Axelborg (Dlg), Vesterbrogade 4A 1503 København V, Denmark. ^b^Milk replacer was produced by Vitfoss, 6300 Graasten, Denmark. ^c^Information about the ingredients and chemical analysis of the milk replacer and creep feed were provided by the producers. ^d^Hemoglobin, 1.05%; monocalcium phosphate, 0.90%; calcium carbonate, 0.82%; calcium formate, 0.80%; vitaLys dry, 0.52%; beet molasses, 0.50%; animal feed salt, 0.46%; threonine, 0.11%; DL-methionine, 0.10%; phytase, 0.04%.

The bacterial challenge strain (*E. coli* O138:F18) was administered in the milk replacer, and was provided to the piglets during the first 3 d after weaning (on day 1, 2, and 3 postweaning). Each day, an individual pig was expected to receive 3 × 10^8^ cfu *E. coli* F18 in 50 mL of milk replacer. The *E. coli* F18 inoculum was prepared according to Sugiharto et al. [12]. The challenge strain was isolated at the Danish Veterinary Institute (Frederiksberg, Copenhagen) from the intestinal contents of a pig with PWD. It was found to carry genes for enterotoxins STb, LT, EAST1, Shigatoxin 2e, and fimbriae F18. The bacterium was hemolytic when grown on blood agar (Oxoid, Deutschland GmbH, Wesel, Germany) [13].

### Sample collection and analysis

For the complete blood counts and immunoglobulin and metabolomic analyses, blood was collected in EDTA-containing and heparinized Vacutainers (Vacuette, Greiner Bio-One GmbH, Kremsmünster, Austria) after puncture of the jugular vein on day 0, 4, and 11 postweaning. Fecal samples were collected for bacterial enumeration and dry matter analysis from the rectum of each piglet with rubber gloves (lubricated with glycerin) on day 0, 2, 3, 4, 5, 7, and 11 postweaning. The diarrhea score, based on the consistency of the collected feces (0 = normal [hard/dry and cloddy/firm/soft with shape] or 1 = diarrhea [soft and liquid/watery and dark/watery and yellow/foamy and yellow]), was recorded. Throughout the experiment, the health (0 = healthy or 1 = unhealthy) and behavior (0 = active or 1 = listless) of the piglets were also evaluated daily. The samples were collected and observations made on day 0 before the piglets were moved to the nursery pen.

To determine the contents of hemolytic *E. coli* and total coliform bacteria, approximately 1 g of feces was suspended in peptone solution (1:10, w/v) and homogenized with a bag mixer (BagMixer100, Interscience, St. Nom, France). Serial 10-fold dilutions were prepared and 100-μL aliquots were plated on blood agar (Oxoid) and McConkey agar (Merck KGaA, Darmstadt, Germany) and incubated aerobically at 37°C overnight to count the hemolytic *E. coli* and total coliform bacteria, respectively. The dry matter content of the feces was determined by freeze-drying the feces samples to a constant weight (in a vacuum at an operating pressure of 0.4 mbar for 48 h). Immediately after collection, complete blood counts were performed with a hemocytometer (Cell-Dyne® 3500R, Abbott Diagnostics, Santa Clara, CA, USA). The plasma was obtained after centrifugation of the blood at 2,000 × *g*, and stored at –20°C until the immunoglobulin and metabolomic analyses. The concentrations of immunoglobulin A (IgA) and immunoglobulin G (IgG) in the plasma were measured with commercial kits (Pig Ig ELISA Quantification Kit, Bethyl Laboratories, Montgomery, TX, USA). The samples were tested at dilutions of 1:5,000 and 1:100,000 for plasma IgA and IgG, respectively. The intra-assay coefficients of variation (CVs) for IgA were 1.7%–2.4% and 0.1%–4.8% for the high- and low-titer control samples, respectively, and the interassay CVs were 3.6% and 4.8%, respectively. For IgG, the intra-assay CVs were 2.6%–3.1% and 1.9%–2.6% for the high- and low-titer control samples, respectively, and the interassay CVs were 2.5% and 2.4%, respectively. The assays were performed in duplicate and according to the manufacturer’s instructions.

The plasma samples were prepared for LC–MS analysis by mixing the plasma (100 μL) with 200 μL of acetonitrile/formic acid (99:1, v/v) containing a 10-μmol/L internal standard mix of glycocholic acid (glycine-1 ^13^C), betaine-trimethyl-d9 hydrochloride, and choline chloride-trimethyl-d9 (Sigma-Aldrich, St. Louis, MO, USA). The samples were shaken and incubated at 4°C for 20 min for deproteinization. The supernatants were collected after centrifugation at 13,200 × *g* for 10 min and injected into the LC–MS apparatus. For the LC–MS analysis, an Ultimate 3000 (Dionex, Sunnyvale, CA, USA) high-performance liquid chromatography (HPLC) system was coupled to a MicrOTOF-Q II mass spectrometer (Bruker Daltonik GmbH, Bremen, Germany) operating in ESI^+^ mode. The scan range was 50–1,000 *m/z*; the capillary voltage was 4,500 V; the nebulizing gas pressure was 1.8 bar; and the drying gas flow and temperature were 8.0 L/min and 200°C, respectively. Lithium formate at a concentration of 5 mmol/L in water/isopropanol/formic acid (50:50:0.2, v/v/v) was used as the external calibrant and was injected at the beginning of each chromatographic run. For subsequent metabolite identification, an MS/MS analysis was performed using argon as the collision gas, with collisions at energies from 6 to 60 eV. Chromatographic separation was performed on an Ascentis® Express HILIC HPLC column (2.7 μm, 2.1 mm × 100 mm) used with a precolumn (2.7 μm, 2.1 mm × 50 mm). The column was held at 30°C and the injection volume was 5 μL. Mobile phase A consisted of ammonium acetate buffer (5 mmol/L at pH 4, adjusted with formic acid) and mobile phase B consisted of acetonitrile/formic acid (100:0.025, v/v). The column was equilibrated for 5 min before the gradient commenced at 90% B for 1 min and then decreased linearly to 10% B within 15 min. The mobile phase was maintained under isocratic conditions (10% B) for 5 min and then returned to 90% B. The total analysis time was 21 min and the flow rate was 100 μL/min. A sample of pooled plasma from the piglets was reinjected after each six samples for quality control of the LC–MS run. The acquired mass spectra were calibrated and converted into mzXML file format using CompassXport (Bruker Daltonik GmbH), before the data were analyzed with XCMS Online, which is freely available at http://xcmsonline.scripps.edu/[14]. The metabolites were identified based on accurate mass and mass spectrometric fragmentation patterns by searching the METLIN databases (http://metlin.scripps.edu/index.php). Standards were used to confirm the identities of the compounds when available.

### Statistical analysis

The effects of the sampling day on the incidence of PWD, health, and behavior of the piglets were analyzed using the GLIMMIX procedure of SAS (SAS Inst. Inc., Cary, NC, USA). The MIXED procedure was used to analyze the effects of the sampling day on the fecal dry matter, bacterial population counts in the feces, concentrations of IgG and IgA in the plasma, and the hematological parameters. A Spearman correlation analysis was used to investigate the relationship between the incidence of PWD, health, and behavior. The individual piglet was considered to be the experimental unit for the statistical evaluations. Litter was included in the model as a random effect. The counts of bacteria (cfu), white blood cells (WBC), and red blood cells (RBC) were log-transformed before the analysis. The data are presented as least-squares means and standard errors of the means (SE). The differences were considered significant when *P* < 0.05.

For the metabolomic data, a principal components analysis and the *P* values for the differences in the intensities of metabolite ions between day 0 and day 4 postweaning were calculated automatically by the XCMS Online system. The false discovery rate (FDR) *q* values, to correct for multiple comparisons, were also calculated automatically by the system, with the significance threshold set at *q* < 0.25 [15].

## Results

### General health of the piglets

**Figure 1:**
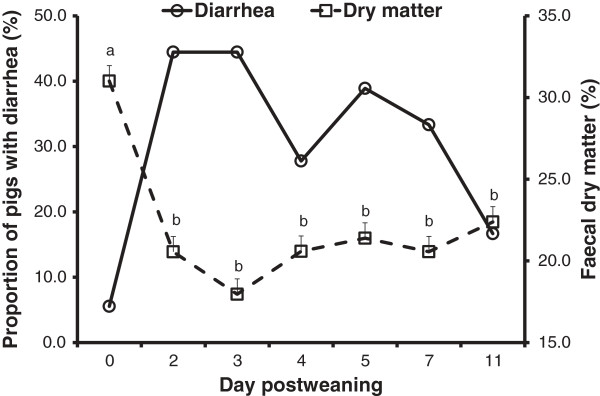
**Proportions of piglets with diarrhea (%) and dry matter in the feces (%) in the days after weaning.** The proportion of piglets with diarrhea increased (*P* = 0.055), whereas the dry matter in the feces decreased (*P* = 0.003) following weaning and challenge with *E. coli*. The piglets were weaned on day 0 and challenged with *E. coli* F18 on day 1, 2, and 3 postweaning. Fecal samples were collected and diarrhea assessed on day 0 before the piglets were moved to the nursery pen.

One of 18 piglets showed signs of diarrhea just before it was moved to the nursery pen, and nearly half (44.4%) the piglets developed diarrhea on day 2 or day 3 postweaning (Figure 1). The dry matter in the feces decreased (*P* = 0.003) after weaning and challenge with *E. coli* (Figure 1). In general, the piglets were healthy and active for the entire experimental period (data not shown). The health of the piglets correlated with their behavior (r = 0.933, *P* < 0.001). The occurrence of diarrhea did not correlate with the health or behavior of the piglets (*P* > 0.05; data not shown).

### Bacterial counts

**Figure 2:**
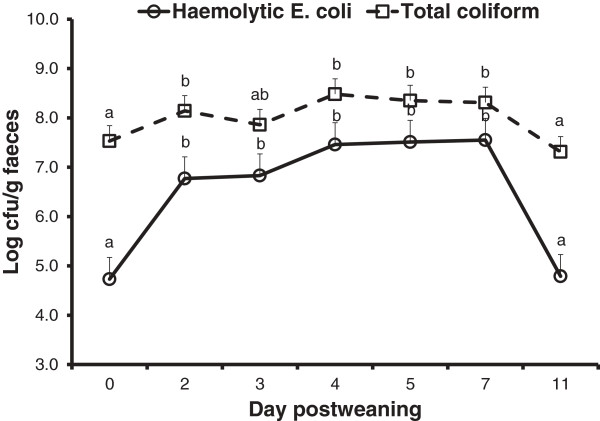
**Fecal shedding of hemolytic *E. coli *and total coliform bacteria (log cfu/g feces) in the postweaning period.** The counts of hemolytic *E. coli* and total coliform bacteria increased (*P* < 0.001) following weaning and challenge with *E. coli*, plateaued, and then declined at the end of experimental period. The piglets were weaned on day 0 and challenged with *E. coli* F18 on day 1, 2, and 3 postweaning.

The counts of hemolytic *E. coli* and total coliform bacteria in the fecal samples increased (*P* < 0.001) following weaning and challenge with *E. coli*, plateaued, and then declined by the end of the experiment (Figure 2).

### Immune responses and hematological parameters

**Figure 3:**
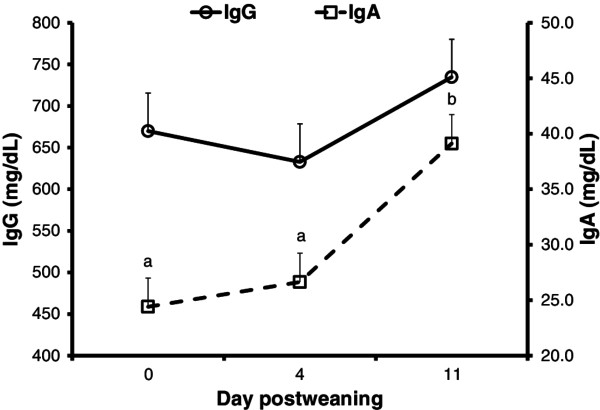
**Concentrations of plasma IgG and IgA (mg/dL) on day 0, 4, and 11 postweaning.** The concentration of IgA was higher (*P* < 0.001) on day 11 postweaning than on day 0 or 4 postweaning. The piglets were weaned on day 0 and challenged with *E. coli* F18 on day 1, 2, and 3 postweaning. Plasma was collected on day 0 before the piglets were moved to the nursery pen.

The concentration of IgA in the plasma was higher on day 11 postweaning than on day 0 or day 4 postweaning (*P* < 0.001; Figure 3). The concentration of plasma IgG did not change during the postweaning period (*P* = 0.158).

The hematological parameters of the piglets are presented in Table 2. The total number of WBC increased continuously from day 0 to day 11 postweaning (*P* < 0.001). The percentage of eosinophils was increased on day 4 postweaning (compared with that on day 0), and then remained unchanged until day 11 postweaning. The percentage of neutrophils and the concentration of hemoglobin did not change from day 0 to day 4 postweaning, but had increased by day 11 postweaning (*P* < 0.05). The percentage of lymphocytes and the hematocrit did not change between day 0 and day 4 postweaning, but had decreased by day 11 postweaning (*P* < 0.05).

**Table 2:** Hematological parameters of piglets on day 0, 4 and 11 postweaning

**Items**	**Day postweaning**^**a**^			**SE**	***P*** **value**
	**0**^**b**^	**4**	**11**		
WBC^c^, × 10^9^/L	10.47^a^	13.80^b^	17.38^c^	<0.01	<0.001
Neutrophils^d^,%	32.49^a^	29.50^a^	39.93^b^	5.42	0.029
Lymphocytes^d^,%	61.00^a^	62.78^a^	52.85^b^	5.23	0.022
NLR^e^	0.62	0.56	0.79	0.14	0.093
Monocytes^d^,%	5.90	6.59	6.12	1.28	0.928
Eosinophils^d^,%	0.35^a^	0.95^b^	0.92^b^	0.19	0.001
Basophils^d^,%	0.11	0.04	0.03	0.04	0.190
RBC^f^, × 10^12^/L	6.61	6.46	6.46	0.00	0.497
Hb^g^, mmol/L	7.27^a^	6.93^a^	11.44^b^	0.37	<0.001
Hematocrit,%	36.50^a^	35.08^ab^	33.75^b^	1.47	0.021

^a^The piglets were weaned on day 0 and challenged with E. coli F18 on day 1, 2, and 3 postweaning. ^b^Blood was collected on day 0 before the piglets were moved to the nursery pen. ^c^WBC, total white blood cells. ^d^The values are presented as percentages of the total WBC. ^e^NLR, neutrophil/lymphocyte ratio; ^f^RBC, red blood cells; ^g^Hb, hemoglobin.

### Metabolomic profile

**Figure 4:**
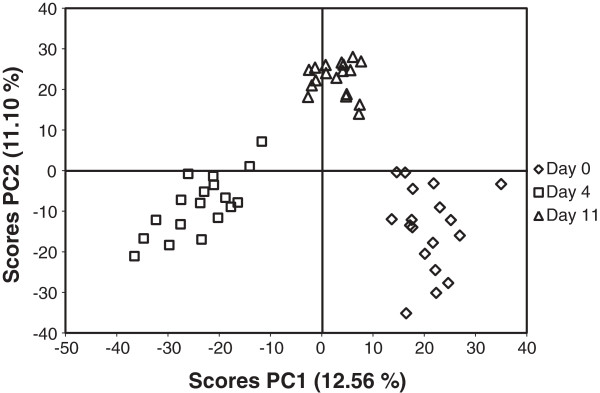
**PCA scores plots of pig plasma collected on day 0, 4, and 11 postweaning.** The PCA model shows a clear separation of the data according to the days postweaning. The piglets were weaned on day 0 and challenged with *E. coli* F18 on day 1, 2, and 3 postweaning. Plasma was collected on day 0 before the piglets were moved to the nursery pen.

A total of 54 plasma samples collected on day 0, 4, and 11 postweaning were included in the metabolomic analysis. The PCA model showed a clear separation of the metabolomic data according to days postweaning. Along the horizontal axis of the PCA (PC1), samples from day 11 postweaning were located between those from day 0 and those from day 4 postweaning (Figure 4).

The metabolite ions with the greatest influence on the differences observed between day 0 and day 4 postweaning were located and identified (Table 3). L-Proline/D-proline, taurine, creatine, carnitine, and L-arginine were identified based on their characteristic fragmentation patterns and retention times. Acetylcarnitine was identified based on its fragmentation pattern, whereas betaine was identified by comparison with a betaine standard. The levels of proline, taurine, and carnitine in the plasma were increased, whereas those of betaine, creatine, L-arginine, and acetylcarnitine were reduced on day 4 postweaning compared with the levels on day 0. Several species of lysophosphatidylcholine (lysoPC) were identified in the plasma and the levels of these metabolites either increased or decreased after weaning, depending on their chain lengths and saturation. The different species of phosphatidylcholine (PC) could not be distinguished in the analysis because their fragmentation patterns were identical. The levels of these compounds were increased or reduced after weaning and challenge with *E. coli*. Of all the metabolites identified in the plasma, an unidentified PC with a mass-to-charge ratio of 678.5 seemed to be the compound most strongly elevated in response to weaning and challenge with *E. coli*.

**Table 3:** **List of metabolites identified in the metabolomic analysis**^***a***^

**Metabolites**	**Retention time, min**	**m/z**	**Mass accuracy, ppm**	**Fold change**^**b**^	**Up/down**	***P*** **value**^**c**^	**FDR q value**^**c**^
L-Proline/D-Proline	9.81	116.0702	3.45	1.35	**↑**	0.0035	0.0046
Betaine	10.93	118.0857	5.08	1.60	**↓**	<0.0001	<0.0001
Taurine	7.15	126.0225	4.76	1.49	**↑**	0.0003	0.0005
Creatine	9.99	132.0760	6.06	3.35	**↓**	0.0002	0.0003
Carnitine	10.78	162.1116	5.55	1.45	**↑**	<0.0001	<0.0001
L-Arginine	11.02	175.1177	7.42	1.82	**↓**	<0.0001	<0.0001
Acetylcarnitine	10.71	204.1218	5.88	1.24	**↓**	0.0003	0.0005
LysoPC 14:0	9.49	468.3054	6.62	3.04	**↑**	<0.0001	<0.0001
LysoPC 16:1	9.39	494.3207	6.88	1.95	**↓**	<0.0001	<0.0001
LysoPC 16:0	9.31	496.3357	8.26	1.20	**↓**	<0.0001	<0.0001
LysoPC 16:0 [M + Na]^+^	9.32	518.3171	8.87	1.19	**↓**	0.0015	0.0021
LysoPC 18:2	9.31	520.3351	9.03	1.90	**↓**	<0.0001	<0.0001
LysoPC 18:0	9.07	524.3667	8.39	1.10	**↓**	0.0428	0.0397
LysoPC 17:0 [K + Na]^+^	9.17	548.3095	3.28	1.40	**↑**	<0.0001	<0.0001
UI PC	8.20	678.5028	5.90	19.75	**↑**	<0.0001	<0.0001
UI PC	8.05	758.5608	11.34	1.43	**↓**	<0.0001	<0.0001
UI PC	7.78	810.5916	11.23	1.31	**↓**	<0.0001	<0.0001
UI PC [M + Na]^+^	7.75	834.5922	7.31	1.26	**↑**	0.0001	0.0003

^a^Discriminating between day 0 and day 4 postweaning. ^b^Fold change was calculated by dividing the mean intensity of each plasma metabolite on day 4 postweaning by the mean intensity of the same plasma metabolite on day 0, calculated automatically by the XCMS Online system. ^c^P values and FDR q values were calculated automatically by the XCMS Online system. UI, unidentified; PC, phosphatidylcholine; LysoPC, lysophosphatidylcholine; **↑**, increased; **↓**, decreased.

## Discussion

Weaning is a stressful event and is associated with intestinal distress and increased exposure to enteric pathogens from feed and the environment [1,4]. To model such stressful conditions, the piglets in this study, which were genetically susceptible to *E. coli* F18, were weaned and challenged with *E. coli* F18. Weaning has been associated with changes of the metabolism of piglets [3,9]. To study the metabolic changes in piglets during the period after weaning, we conducted an LC–MS-based metabolomic analysis of the plasma from piglets, collected at different time points after weaning. As shown in the PCA scores plot, there was a separation between the metabolic profiles according to the days after weaning, indicating changes in the metabolic patterns of the piglets during the postweaning period. The first week (5–7 d) after weaning is considered the most stressful period for piglets, when intestinal dysfunction and changes in the metabolism can occur [3,11]. Consistent with this assumption, a clear separation was observed in the serum metabolites between day 0 and day 4 postweaning in the PCA model. Interestingly, the samples from day 11 postweaning were located between those from day 0 and day 4 postweaning along the horizontal axis of the PCA (PC1). This observation suggests that the piglets on day 11 postweaning tended to resume a metabolic state similar to that observed on day 0. In this study, we noticed an increase in the incidence of PWD and fecal shedding of hemolytic *E. coli* during the first week postweaning, but these parameters had decreased on day 11 postweaning. Based on the pattern of metabolic changes indicated by the PCA model and the development of PWD and fecal shedding of hemolytic *E. coli*, we inferred a potential relationship between the metabolomic profiles of piglets and weaning- and *E. coli*-induced PWD during the early postweaning period.

In this study, we noted that the responses of some blood immune parameters to weaning and *E. coli* challenge were variable and dynamic. Whereas no difference was observed in plasma IgG in the days after weaning, the plasma IgA concentrations increased with weaning and *E. coli* F18 challenge. It has been reported that physiological and immunological changes occur with age and weaning [2,16]. In this context, we realize that our failure to include a control group (piglets not challenged with *E. coli* F18) in the present study limits our interpretation of the results, and that developmental changes may have contributed to some of the changes observed. Previous studies have shown that *E. coli* F18 infection enhances IgA levels in the sera and intestinal washes of postweaning piglets [17], and that once IgA has been stimulated (by pathogens), it may persist for a long period (the half-life of IgA is 6 d [18]). Therefore, we inferred that the enhanced IgA concentration was primarily attributable to the *E. coli* F18 challenge, because weaning is normally associated with a reduced concentration of IgA (with the removal of the IgA derived from the sow’s milk) [4]. The fact that we weaned the piglets at 28–34 d of age may also support our inference, because it is assumed that pigs are physiologically and immunologically competent by 35 d of age [16].

Our results show that the number of total WBC continued to increase from day 0 to day 4 and 11 postweaning. This is consistent with a study of unchallenged postweaning piglets (weaned at 21 d of age) by Davis et al. [19], which showed increased total WBC following weaning (and age), i.e., 3.51, 3.92, 6.53, and 8.82 × 10^3^/mL on day 0, 2, 10, and 27 postweaning, respectively. Therefore, it seems difficult to attribute the increased total WBC in our piglets to the effects of the stress induced by weaning and challenge with *E. coli*. Moreover, although differences in the percentages of neutrophils and lymphocytes were seen in the postweaning period, the ratio of neutrophils to lymphocytes (an indicator of the pig’s response to stress) did not change during this period, suggesting that the observed differences in the percentages of neutrophils and lymphocytes were probably age-related and were not due to weaning and challenge with *E. coli*[19].

In the metabolomic analysis, seven lysoPC species were identified in the plasma of pigs and their levels changed during the experiment, with two species increasing and five species decreasing when the piglets were weaned and challenged with *E. coli*. LysoPC is a bioactive phospholipid involved in inflammatory processes [20]. Therefore, the elevated levels of the two lysoPC species in the plasma of postweaning pigs were probably related to weaning- and *E. coli*-induced inflammation and the development of PWD [2,4], which were apparent as increased fecal shedding of hemolytic *E. coli* and the occurrence of PWD during the first week after weaning in this study. LysoPC is also known as an important intermediate in the degradation and biosynthesis of PC [21]. In this study, we observed a substantial increase in the levels of a PC with a mass-to-charge ratio of 678.5 in the plasma (an indicator of increased PC formation) in the postweaning pigs. This increase in PC biosynthesis might consequently reduce the availability of some particular lysoPC species in the circulatory system, and may partly explain the reductions in five lysoPC species in the plasma of postweaning pigs in the present study. Increased gut colonization by ETEC is commonly seen in postweaning pigs [4], and accordingly, increased fecal shedding of hemolytic *E. coli* was observed in our pigs after weaning. ETEC colonization may increase the production and/or expression of proinflammatory molecules in the pig gut [2,4], and these molecules can react with free radicals, leading to cell membrane damage [22]. Because PC is the major phospholipid component of mammalian cell membranes [23], the increased formation of the two particular PCs observed in this study is most reasonably ascribed to an increased need in these PCs for membrane biogenesis during the early postweaning period. We also observed two unidentified species of PC in the plasma, the levels of which decreased. The reduction in these plasma PCs was probably associated with the enhanced degradation of some specific type of PC during the inflammation-related processes around weaning [24,25], resulting in the increased levels of particular lysoPC species (as described above). Taken together, these data suggest that PC and lysoPC respond differently to weaning and exposure to pathogens, depending on their chain lengths or the molecular characteristics of these metabolites.

Betaine, creatine, and l-arginine have been reported to confer protective effects against oxidative stress [26-28]. In our study, the plasma levels of these compounds decreased upon weaning and exposure to *E. coli*. The inhibition of the antioxidant system, leading to an inflammatory state and the development of PWD in pigs after weaning [29], might partly explain this circumstance. In this study, the increase in carnitine was observed in the plasma of pigs after weaning. Carnitine is an important compound with roles in the intermediary metabolism. Thus, generating adequate levels of carnitine could be one attempt by the piglets to prevent metabolic disturbance and maintain normal metabolic functions during the period after weaning [30]. In contrast to carnitine, the level of acetylcarnitine, an acetyl derivative of carnitine and a byproduct of mitochondrial β-oxidation, decreased after weaning. Zhao et al. [31] reported that the levels of plasma acylcarnitine may indicate the extent of fatty acid β-oxidation and the energy status of animals. However, the reduction in plasma acylcarnitine remains unexplained in this study because we did not measure the feed intake of the piglets throughout the experiment. The plasma levels of proline increased noticeably in the piglets after weaning. The upregulation of proline biosynthesis seems to be a response mechanism of piglets to the stress and/or oxidative stress induced by weaning and exposure to *E. coli*[32], apparent as an increased fecal content of hemolytic *E. coli* and an increased incidence of PWD. Taurine is one of the most abundant free amino acids in animals and is present at high concentrations in WBC [33]. In this study, the increased plasma levels of taurine paralleled the increases in the total WBC concentration. Hence, the observed increased production of taurine in the piglets after weaning does not seem to be associated with weaning stress, but instead supported the development of the pig immune system as the pigs grew older.

## Conclusions

Our results showed a clear separation in the plasma metabolomic profiles of pigs in the days after weaning that corresponded to the fecal response to stresses on the piglets, i.e., weaning and exposure to pathogens (*E. coli*). Based on the plasma metabolite profiles, these challenges might induce proinflammatory responses in piglets. The challenges also induced PWD and increased the concentration of IgA in the plasma of piglets.

## Competing interests

The study was partly funded by Arla Foods Amba, Denmark, and partly by a PhD scholarship received from the Directorate General of Higher Education, Ministry of Education and Culture, Republic of Indonesia. The authors declare that they have no conflicts of interest.

## Authors’ contributions

SS performed the animal study and drafted the overall manuscript; MSH performed the metabolomic analysis and revised the drafted manuscript; and CL was the project coordinator, designed the experiment, and revised the drafted manuscript. All authors read and approved the final manuscript.
